# Percutaneous Coronary Intervention versus Coronary Artery Bypass Grafting for Non-Protected Left Main Coronary Artery Disease: 1-Year Outcomes in a High Volume Single Center Study

**DOI:** 10.3390/life12030347

**Published:** 2022-02-27

**Authors:** Ștefan Dan Cezar Moț, Adela Mihaela Șerban, Ruxandra Ștefana Beyer, Mihai Cocoi, Horia Iuga, Ioana Dănuța Mureșan, Simona Cozma, Alexandra Dădârlat-Pop, Raluca Tomoaia, Dana Pop

**Affiliations:** 1 Cardiology Department, Heart Institute “N. Stăncioiu”, 400001 Cluj-Napoca, Romania; adelamserban@elearn.umfcluj.ro (A.M.Ș.); anda_bogdan@yahoo.com (R.Ș.B.); mihai.cocoi@umfcluj.ro (M.C.); iuga.horia@gmail.com (H.I.); dadarlat.alexandra@elearn.umfcluj.ro (A.D.-P.); 2 5th Department of Internal Medicine, Faculty of Medicine, “Iuliu Hațieganu” University of Medicine and Pharmacy, 400012 Cluj-Napoca, Romania; ioanamuresandanuta@elearn.umfcluj.ro (I.D.M.); dana.pop@elearn.umfcluj.ro (D.P.); 3 Department of Cardiology, Ares Hospital, 400015 Cluj-Napoca, Romania; simo.cozma@gmail.com; 4 Department of Cardiology, Clinical Rehabilitation Hospital, 400347 Cluj-Napoca, Romania

**Keywords:** left main, percutaneous coronary intervention, coronary artery bypass grafting, mortality

## Abstract

Introduction: There is clear evidence of a significant reduction in all major cardiovascular adverse events (MACE) by coronary artery bypass grafting (CABG) in left main coronary artery stenosis (LMCS), but revascularization by percutaneous coronary artery intervention (PCI) shows an increasingly important role as an alternative to CABG. Several recent trials aiming to test the difference in mortality between the two types of revascularization found conflicting data. The aim of this study is to determine whether PCI is non-inferior to CABG with respect to the occurrence of MACE at 1 year in patients with significant LMCS. Material and methods: We prospectively enrolled 296 patients with chronic or acute coronary syndromes and significant LM stenosis. The angiography that recommended the revascularization procedure was used for the calculation of the Syntax II score, in order to classify the patients as low-, intermediate- or high-risk. Low- and high-risk patients were revascularized with either PCI or CABG, according to current guidelines, and were included in the subgroup S1. The second subgroup (S0) included intermediate-risk patients (Syntax II score 23–32), in whom the type of revascularization was chosen depending on the decision of the heart team or the patient preference. Patients were monitored according to the chosen mode of revascularization—PCI or CABG. LM revascularization was performed in all the patients. Clinical endpoints included cardiac death, myocardial infarction, need for revascularization and stroke. Patients were evaluated at 1 year after revascularization. Event rates were estimated using the Kaplan–Meier analysis in time to the first event. Results: At 1-year follow-up, a primary endpoint occurred in 35/95 patients in the CABG group and 37/201 in the PCI group. There were no significant differences between the 2 treatment strategies in the 1-year components of the end-point. However, a tendency to higher occurrence of cardiac death (HR = 1.48 CI (0.55–3.9), *p* = 0.43), necessity of repeat revascularization (HR = 1.7, CI (0.81–3.6), *p* = 0.16) and stroke (HR = 1.52, CI (1.15–2.93), *p* = 0.58) were present after CABG. Contrariwise, although without statistical significance, MI was more frequent after PCI (HR = 2, CI (0.78–5.2), *p* = 0.14). The Kaplan–Meier estimates in subgroups demonstrated the same tendency to higher rates for cardiac death, repeat revascularization and stroke after CABG, and higher rates of MI after PCI. Although without statistical significance, patients with an intermediate-risk showed a slightly lower risk of MACE after PCI than CABG. With the exception of dyslipidemia and gender, other cardiovascular risk factors were in favor of CABG (CKD, obesity). Conclusion: In patients with LMCS, PCI with drug-eluting stents was non-inferior to CABG with respect to the composite of cardiac death, myocardial infarction, repeat revascularization and stroke at 1 year, even in patients with intermediate Syntax II risk score.

## 1. Introduction

Left main coronary artery stenosis (LMCS) is detected in 5–7% of all diagnostic coronary angiograms [1] and is of particular importance because it reduces up to 84% of left ventricular (LV) vascularization in patients with right coronary artery (CA) dominance and 100% in those with left CA dominance [2]. There is clear evidence of a significant reduction in all major cardiovascular adverse events (MACE) by coronary artery bypass grafting (CABG) [3]. However, recent data focused on the improved efficacy and safety of percutaneous cardiovascular interventions (PCI), granting PCI an increasingly important role [4].

Several trials aiming to test the difference in mortality between the two types of revascularization found conflicting data. PRECOMBAT and SYNTAX trials [5,6,7,8] found similar mortality between PCI and CABG, while the EXCEL trial [9] found higher mortality after PCI at 5-years follow-up. The NOBLE trial demonstrated similar mortality between the two types of revascularizations, but with the inferiority of PCI due to higher rates of non-procedural myocardial infarction and repeat revascularization [10,11].

Applying risk scores guides the selection between the two types of revascularization by performing the estimation of the intermediate to long-term prognosis. The Syntax II score combines the Syntax I anatomical score with three clinical variables (age, presence of renal failure, and reduced LVEF), each known as a direct indicator of influence of mortality. This score was described in the Delta registry [12] and was also validated by the Excel trial [9]. Therefore, current guidelines recommend revascularization with either PCI or CABG in low-risk patients (Syntax II score ≤ 22, class of recommendation I) and with CABG in high-risk patients (Syntax II score > 32, class of recommendation I), whereas intermediate-risk patients (Syntax II score 23–32), have a stronger recommendation for CABG (class I) than PCI (class IIA) [13].

However, improvement of interventional techniques in recent years may lead to an even greater decrease of MACE after PCI and a similar prognosis between the two types of revascularization.

The aim of this study is to determine whether PCI is non-inferior to CABG with respect to the occurrence of MACE at 1 year in patients with significant LMCS. 

## 2. Materials and Methods

### 2.1. Study Population

This research was conducted in accordance with the Declaration of Helsinki and was approved by the Ethics Committee of “Nicolae Stăncioiu” Heart Institute, Cluj-Napoca, Romania, number 17/2017 It was a single-center study, which prospectively enrolled 304 patients who were admitted in the cardiology department from 2018 to 2020. Inclusion criteria were the following: chronic (stable angina pectoris, dyspnea) and acute coronary syndromes (unstable angina pectoris, NSTEMI, STEMI), significant stenosis (with a visually assessed diameter of stenosis >70% or FFR ≤ 80% in case of a visually assessed stenosis of 50–70%) in the LM coronary artery (ostium/ mid-shaft/ bifurcation). Exclusion criteria included severe comorbidities with an expected survival of less than 1 year and prior CABG. Cardiac enzymes, 12-lead electrocardiogram and echocardiography, including left ventricular ejection fraction (LVEF) were performed after admission in all the patients. All patients offered their written informed consent.

All patients underwent coronary artery angiography. The angiography that recommended the revascularization procedure was analyzed and used for the calculation of the Syntax II anatomical and clinical score in order to classify the patients as low- (≤22), intermediate- (23–32) or high-risk (>32). Patients were monitored according to the chosen mode of revascularization—PCI or CABG.

A heart team, including an interventional cardiologist and a heart surgeon, assessed all the patients for eligibility for either percutaneous or surgical treatment.

LM coronary artery revascularization was performed in all the patients. Low-risk patients (Syntax II score ≤ 22) were revascularized with either PCI or CABG (according to current recommendations, to the heart team decision depending on the anatomical complexity and according to the patient’s preference); high-risk patients (Syntax II score > 32) were revascularized with CABG, according to current guidelines [12]; both low- and high-risk patients were included in the subgroup S1, where the type of revascularization had a class I indication in the current guidelines. The second subgroup (S0) included intermediate-risk patients (Syntax II score 23–32), in whom the type of revascularization was chosen, depending on the decision of the heart team or the patient preference, where CABG has a class I indication and PCI a class IIA indication in the current European Guidelines on myocardial revascularization [12].

### 2.2. Procedures

The goal of both PCI and CABG was the complete revascularization of all ischemic territories. PCI was performed by using only drug-eluting stents. Distal LM bifurcation stenoses were treated with one or two-stent techniques and guidance with intravascular ultrasonography was used when considered necessary by the operators. Kissing balloon dilatation was mandatory when using the two stents technique or at the discretion of the operator when using a single-stent technique.

CABG was performed with the use of arterial grafts, when possible, but venous grafts were also be used. 

All the participants received medical treatment after the procedure according to current recommendations. Aspirin was administered lifelong in both groups and a P2Y12 inhibitor was associated after PCI for 6 to 12 months, according to the type of clinical presentation of admission. 

### 2.3. Follow-Up

The study was designed in order to determine if PCI was non-inferior to CABG in reference to the occurrence of MACE at 1 year. Clinical endpoints included cardiac death, myocardial infarction, need for revascularization and stroke. Patients were evaluated by outpatient control, and telephone or hospital records were used to evaluate patients at 1 year after revascularization.

### 2.4. Statistical Analysis

Clinical variables were expressed as mean ± SD or frequencies depending on the type and distribution. Normality was tested via the Kolmogorov–Smirnov test. Patients were divided into two groups according to the type of revascularization (PCI vs. CABG). Continuous data were compared using *t*-tests if there was a normal distribution or Mann–Whitney test otherwise, and categorical data by using the Chi^2^ test or Fisher’s test. 

Follow-up began after revascularization and continued up to 1 year or until the occurrence of the first MACE. We first evaluated the occurrence of all MACE and of each separate end-point in the whole group of patients. Secondly, the occurrence of MACE was analyzed separately in two subgroups formed on the basis of the Syntax II score (S0 and 1). The first subgroup (S1) included patients in whom the type of revascularization was chosen according to current recommendations (low-risk patients were treated with either PCI/CABG (Syntax II score ≤ 22) and high-risk patients were treated with CABG (Syntax II score > 32)). The second group (S0) included intermediate-risk patients (Syntax II score 23–32), in whom the type of revascularization was elected depending on other variables. Clinical follow-up in all patients and, according to the S0/1 subgroups, was reported using the Kaplan–Meier analysis, 95%CI and the log-rank test. 

Subgroups analyses performed for the variables that showed significant differences between groups (gender, dyslipidemia, obesity, CKD, acute coronary syndrome on presentation, complete revascularization and S0) were presented using a forest plot and hazard ratios, 95% CI and interactions tests. The *p* values for interaction were obtained with likelihood ratio tests.

Statistical analysis was performed with MedCalc Statistical Software 19.6.1 (MedCalc Software Ltd., Ostend, Belgium; http://www.medcalc.org; (accessed on 22 Janaury 2022)). A *p* value of <0.05 was considered significant. 

## 3. Results

### 3.1. Baseline Characteristics

**Figure 1:**
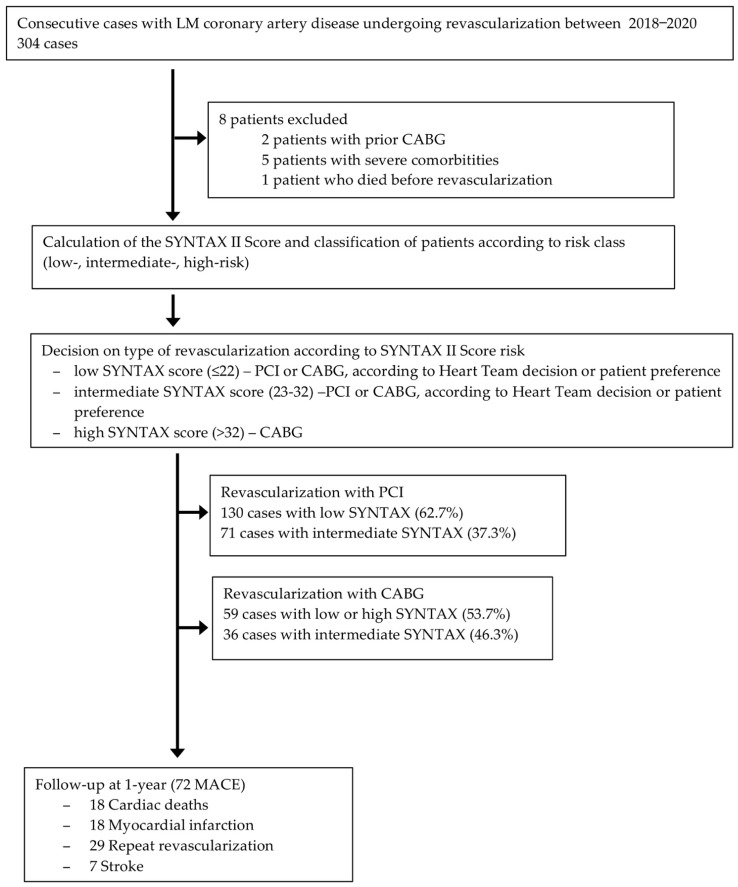
Study flowchart. CABG, coronary artery bypass grafting; LM, left main; MACE, major cardiac adverse events; MI, myocardial infarction; PCI, percutaneous coronary intervention.

Between 2018 and 2020, 304 patients with significant LM coronary artery disease were recruited (Figure 1); 8 patients were excluded (2 patients with prior CABG, 5 patients with severe comorbidities (e.g., cancer) and 1 patient who died before revascularization). The SYNTAX II score was calculated for all the remaining patients and was used to classify them according to the resulting risk, into low- (≤22), intermediate- (23–32) and high-risk (>32) classes. All patients underwent LM coronary artery revascularization.

There were 130 patients in the low-risk class, who underwent PCI and 59 low- or high-risk patients who underwent CABG (according to the heart team’s decision, depending on the anatomical complexity and according to the patient’s preference) and 59 patients in the high-risk class, which were revascularized by CABG. Regarding patients with an intermediate-risk, the decision on the type of revascularization was made according to the heart team, or to the patient preference. There were 107 intermediate-risk patients, 71 of whom were referred for PCI and 36 for CABG.

At the 1-year follow-up, there were 72 MACE: 18 cases of cardiac death, 18 cases of myocardial infarction, 29 cases of recurrent angina requiring repeat revascularization, and 7 cases of stroke.

Baseline characteristics of patients undergoing revascularization with PCI (n = 201, 68%) and CABG (n = 95, 32%) are shown in Table 1. Patients undergoing PCI presented more cardiovascular risk factors (dyslipidemia, obesity and chronic kidney disease) and were more frequently admitted with acute rather than chronic coronary syndromes (*p* < 0.0001) compared with subjects undergoing CABG. 

Patients in the CABG group presented more frequently with multivessel CAD (79% vs. 52.2%, *p* < 0.0001), but there was no significant difference regarding the number of coronary arteries with significant stenosis between the two groups (chi^2^p = 0.09). Complete revascularization was more frequently encountered with PCI (68.7% vs. 53.7%, *p* = 0.012). We also experienced a less extensive use of arterial conducts in the CABG group.

On the 1-year follow-up, patients undergoing GABG presented significantly more MACE than patients with PCI (35/95 patients, 36.8% vs. 37/201 patients, 18.4%). Cardiac death, repeat revascularization and stroke were more frequent after CABG, while MI occurred more frequently after PCI.

### 3.2. Major Cardiac Adverse Events (MACE)

**Figure 2:**
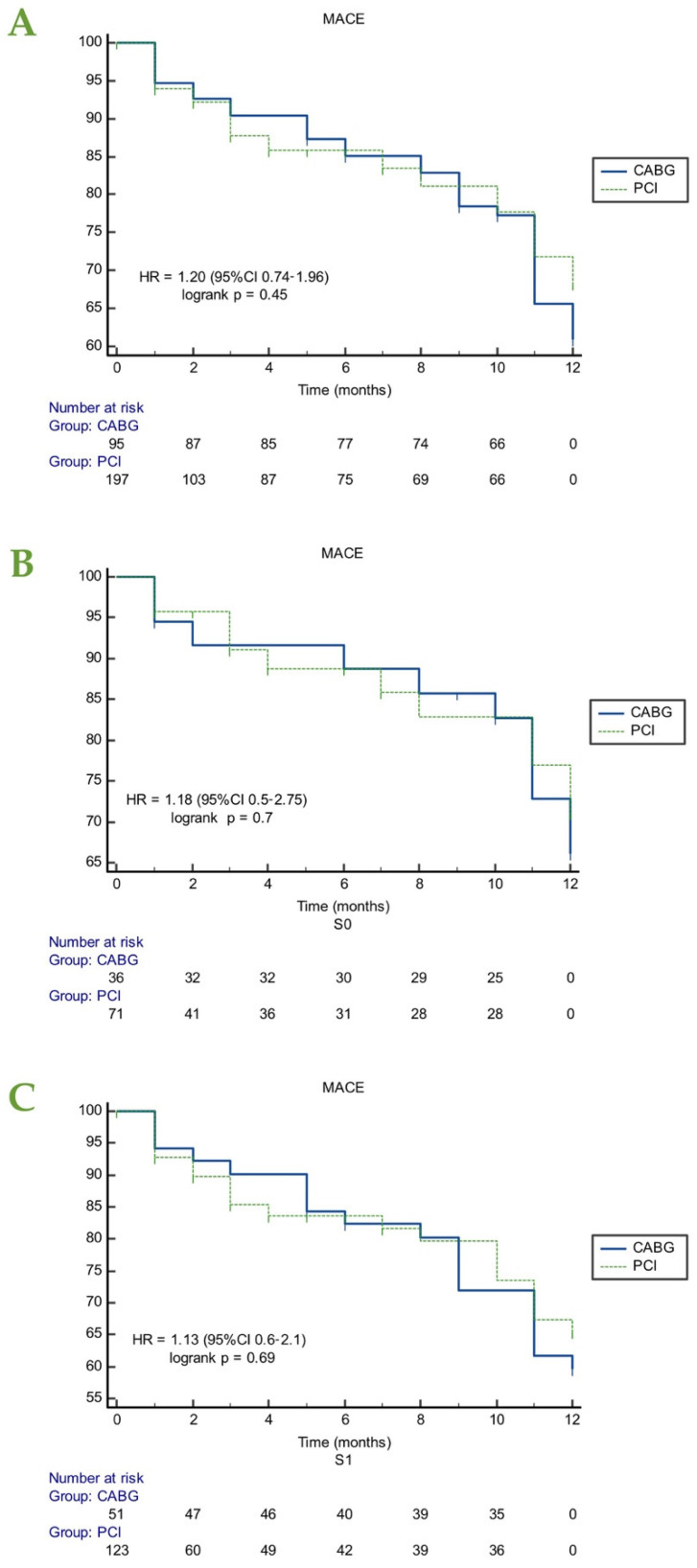
Kaplan–Meier estimates of MACE according to the type of invasive treatment strategy (**A**) in all patients, (**B**) when the choice of invasive treatment between PCI and CABG was performed according to the decision of the heart team/ patient preference (S0) and (**C**) when the choice of invasive treatment between PCI and CABG was performed in accordance with current guidelines (S1). CABG, coronary artery bypass grafting; MACE, major cardiac adverse events; PCI, percutaneous coronary intervention.

**Figure 3:**
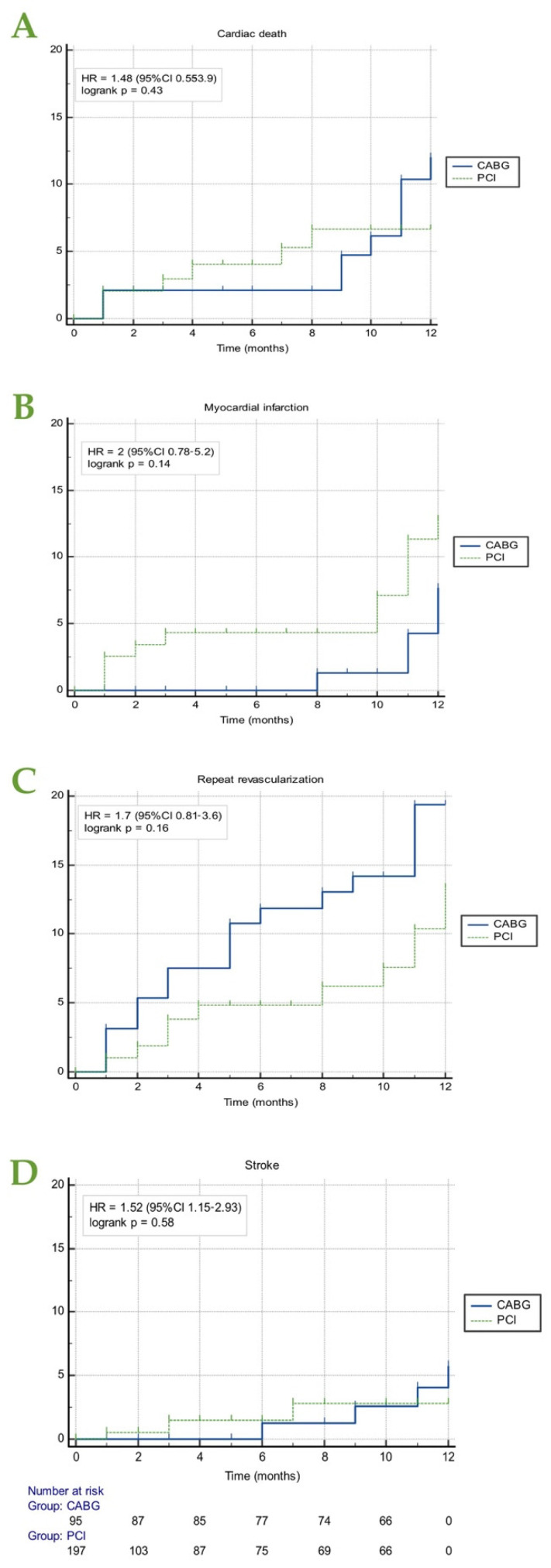
Kaplan–Meier estimates of each end-point (**A**) cardiac death, (**B**) myocardial infarction, (**C**) repeat revascularization, (**D**) stroke) according to the type of invasive treatment strategy in all patients. The number at risk is that reported for all types of MACE. CABG, coronary artery bypass grafting; MACE, major cardiac adverse events; PCI, percutaneous coronary intervention.

**Figure 4:**
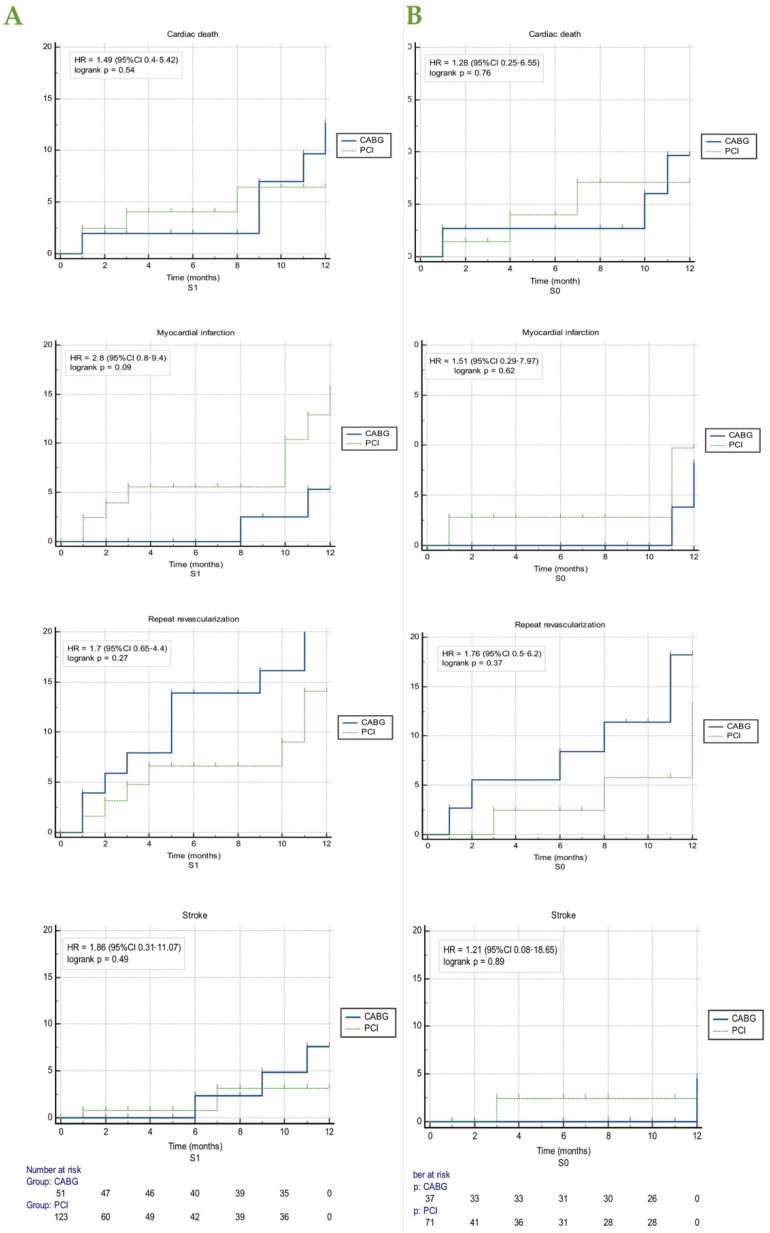
Kaplan–Meier estimates of each end-point according to the type of invasive treatment strategy, (**A**) when the choice of invasive treatment between PCI and CABG was performed in accordance with current guidelines (S1) and (**B**) when the choice of invasive treatment between PCI and CABG was performed according to the decision of the heart team/patient preference (S0). The number at risk is that reported for all types of MACE, according to each subgroup. CABG, coronary artery bypass grafting; MACE, major cardiac adverse events; PCI, percutaneous coronary intervention.

The duration of follow-up was 1 year in both groups. The occurrence of any type of MACE according to the type of revascularization (PCI vs. CABG) in all the patients and in compliance to the risk group patients were assigned to, are presented in Figure 2. The occurrence of each end-point after PCI vs. CABG, in all the patients and in subgroups are shown in Figure 3 and Figure 4. The end-point event of cardiac death, MI, repeat revascularization or stroke occurred in 18.4% of the patients in the PCI group and 36.8% of the patients in the CABG group (*p* = 0.0006).

The 1-year rates of any type of MACE were not significantly different between groups. However, there was a tendency towards a higher occurrence of MACE after CABG, in both patients assigned as S1 (estimated rate of 13%, HR = 1.13 (CI 0.6–2.1), *p* = 0.69) and S0 (estimated rate of 18%, HR = 1.18, CI (0.5–2.75), *p* = 0.7).

There were no significant differences between the two treatment strategies in any of the 1-year components of the end-point (cardiac death, MI, repeat revascularization and stroke)—Figure 3. Yet, a tendency to higher occurrence of cardiac death (HR = 1.48, CI (0.55–3.9), *p* = 0.43), necessity of repeat revascularization (HR = 1.7, CI (0.81–3.6), *p* = 0.16) and stroke (HR = 1.52, CI (1.15–2.93), *p* = 0.58) was also present after CABG. Contrariwise, although without statistical significance, MI was two times more frequent after PCI than CABG (HR = 2, CI (0.78,–5.2), *p* = 0.14).

The Kaplan–Meier estimates of the endpoints according to the subgroup patients were assigned to (S0 vs. 1) and demonstrated the same tendency towards higher rates after CABG for cardiac death (HR = 1.28, CI (0.25–6.55), *p* = 0.76 and HR = 1.49, CI (0.4–5.42), *p* = 0.54), repeat revascularization (HR = 1. 76, CI (0.5–6.2), *p* = 0.37 and HR = 1. 7, CI (0.65 to 4.39), *p* = 0.27) and stroke (HR = 1.21, CI (0.08–18.65), *p* = 0.89, and HR = 1.86, CI (0.31–11.07), *p* = 0.49,) and of MI after PCI (HR = 1.51, CI (0.29–7.97), *p* = 0.62 and HR = 2.8 (0.8–9.4) *p* = 0.09) (Figure 4).

**Figure 5:**
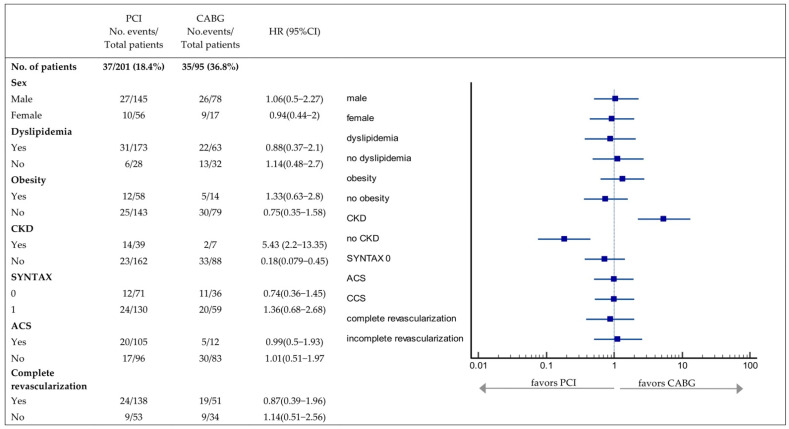
Forest plot of MACE according to subgroups. HR is calculated for the end-point of cardiac death, repeat revascularization, myocardial infarction and stroke. The *p* values for interaction were obtained by likelihood ratio tests between each variable and the treatment. ACS, acute coronary syndrome; CABG, coronary artery bypass grafting; CKD, chronic kidney disease; MACE, major cardiac adverse events; PCI, percutaneous coronary intervention.

The outcomes after each type of revascularization are shown in Figure 5. There was no significant interaction between any of the subgroups. Although without statistical significance, patients with an intermediate-risk (S0) showed a slightly lower risk of MACE after PCI than CABG. With the exception of dyslipidemia and gender, other cardiovascular risk factors were in favor of CABG (CKD, obesity).

### 3.3. Left Ventricular Ejection Fraction

**Figure 6:**
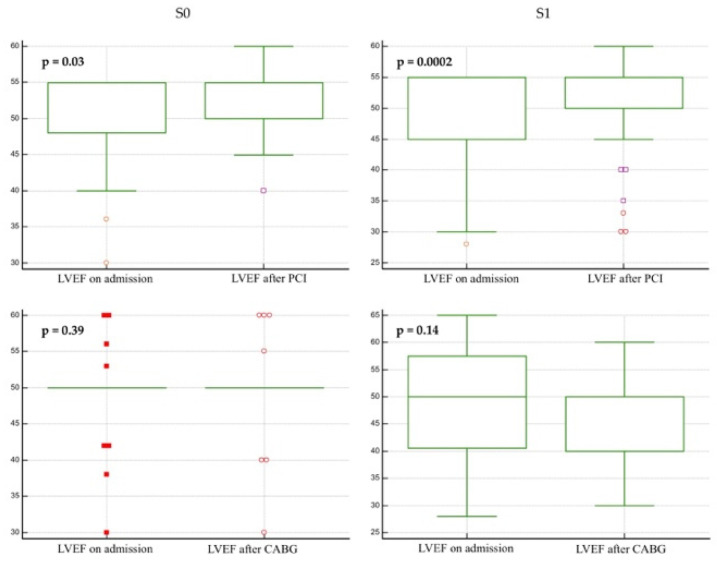
Difference between LVEF on admission and on follow-up according to each type of procedure and S0/1. CABG, coronary artery bypass grafting; LVEF, left ventricular ejection fraction; PCI, percutaneous coronary intervention.

LVEF before and after each type of procedure was used to evaluate the effect of revascularization on LV systolic function. There was a statistically significant recovery of the LVEF in both S1 (49.7 ± 7.5 vs. 52 ± 6.5, *p* = 0.0002) and S0 patients (50.9 ± 6 vs. 52.5 ± 4.2, *p* = 0.03), which was not present also after CABG (Figure 6).

## 4. Discussion

LMCS represents a challenge for cardiovascular medical and surgical teams. Most patients with significant LMCS are symptomatic and at high-risk of MACE. In the absence of revascularization, survival at 3 years is about 37% [14]. Although CABG has a long history of efficacy and safety, recent meta-analyses suggest similar developments by applying percutaneous intervention techniques [15,16,17,18]. Modern surgical and interventional revascularization techniques compete to improve morbidity and mortality in LMCS disease. The standard of treatment for the last 20 years—coronary artery bypass grafting—has evolved through the use of arterial grafts, off-pump or minimally invasive techniques, which have made it attractive to practitioners and patients. On the other hand, percutaneous coronary interventions have developed exponentially, with the introduction of pharmacologically active stents, adjuvant therapy, intracoronary imaging, but especially through the improvement of interventional cardiology techniques [13]. This constantly changing field requires continuous comparisons, correlations and adjustments, based on scientifically proven experience. The goal of our study was to determine whether PCI is non-inferior to CABG with respect to the occurrence of MACE at 1 year in patients with significant LMCS, and we found that there were no significant differences between the two treatment strategies in the 1-year components of the end-point (cardiac death, MI, repeat revascularization and stroke). 

Among recent trials, EXCEL [9] and NOBLE [10,11] best compare the two types of revascularization, showing that there are similar rates of death from any cause, myocardial infarction and stroke at over a year. Patients with simpler coronary anatomy benefit more from PCI, which is the preferred way to revascularize some subtypes of patients. The more complex the anatomy and the higher the syntax score, there is the growing benefit of CABG. It should be noted that MACE are more common with GABG during hospitalization, but during follow-up, the rate of MACE equalizes between CABG and PCI, with the exception of myocardial infarction and of the need for repeated revascularization, which is higher with PCI [10,19]. However, in these last trials, there was no sub-analysis on the clinical criteria for hospitalization (acute vs. chronic coronary syndromes). In our study, although there were no significant differences between the two treatment strategies regarding MACE, there was a tendency to a higher occurrence of myocardial infarction after PCI and of cardiac death, repeat revascularization and stroke after CABG. This tendency was less pronounced in patients with an intermediate-risk (Syntax II score of 23–32%), where the estimated rates demonstrated smaller differences between the two types of revascularization. A recent meta-analysis also demonstrated similar safety between the two types of revascularization in patients with LMCS and low to intermediate complexity CAD, but repeat revascularization occurred more frequently after PCI [15,20]. Although previous studies demonstrated an advantage of CABG over PCI in patients with multivessel coronary artery disease [21], our results showed lower completeness of revascularization rates in the CABG group. The different results compared to those of other studies might be explained by a greater extent of CAD in this group. Furthermore, compared to the PCI group, the CABG group in our study included a higher number of high-risk patients, in whom the possibility of complete revascularization was reduced. Another explanation for the lower completeness of revascularization in the CABG group might be related to the less extensive use of arterial grafts, which has been observed in our study. 

The subgroups’ analyses did not particularly suggest that patients with intermediate Syntax II scores are more suitable for CABG or PCI, which is consistent with the findings in the EXCEL trial [9]. In our study, the clinical presentation with ACS showed a similar outcome after PCI and CABG.

Patients undergoing PCI presented with more cardiovascular risk factors and were more frequently admitted with acute rather than chronic coronary syndromes compared to subjects undergoing CABG. When separate clinical factors were analyzed according to the type of revascularization strategy, patients with an intermediate-risk (S0) showed a similar risk of MACE after both PCI and CABG, with slightly lower risk values for PCI. With the exception of dyslipidemia and gender, other cardiovascular risk factors were in favor of CABG (chronic kidney disease, obesity). In order to evaluate the LV systolic function after revascularization, LVEF was measured before and after revascularization. There was a statistically significant recovery of the LVEF in both S1 and S0 patients, which was not present after CABG. However, although statistically significant, the difference was not clinically relevant (<2.3%).

Overall, our results demonstrate the non-inferiority of PCI compared to CABG with regard to the occurrence of MACE at 1 year after revascularization, with a tendency to higher rates of myocardial infarction after PCI. Our results are similar to the PRECOMBAT and SYNTAX trials [5,6,7,8], which also found similar mortality between PCI and CABG at 5 years. Furthermore, the NOBLE trial demonstrated similar mortality between the two types of revascularization, but PCI was inferior to CABG due to higher rates of non-procedural myocardial infarction and repeat revascularization [10,11]. In contrast, other trials (EXCEL and updated 5-year outcomes from the NOBLE trial) [11,19] found higher mortality after PCI at the 5-years follow-up. 

Altogether, the choice of type of revascularization necessitates discussions between the patient, his relatives and the complex medical team (clinical cardiologist, interventional cardiologist, cardiovascular surgeon, anesthesiologist, and sometimes other specialists) [22,23,24]. We emphasize that the CABG associated higher risk of stroke and longer recovery and might warrant the choice of PCI in patients eligible for both revascularization strategies. Moreover, the majority of the patients with low- or intermediate-risk in this study opted for PCI, which is a less invasive technique when compared to CABG. We, therefore, highlight the importance of individualized therapy and consideration of patient preference in the choice of revascularization. 

This study also presents several limitations. First, it was a single-center study. Secondly, long-term medication was different between patients, and this might affect the impact on MACE occurrence. Thirdly, the follow-up duration was relatively short and longer periods of monitoring might be necessary to evaluate supplementary differences between revascularization strategies. Furthermore, we acknowledge that the choice between PCI and CABG, according to the local expertise and to the clinical and anatomical characteristics, might be of subjective matter and also a major limitation of the study. However, these aspects must be taken into account and the choice made individually for each patient. Further studies should use a longer follow-up duration to determine if PCI is an alternative for CABG in intermediate-risk patients.

## 5. Conclusions

In conclusion, in patients with LMCS, PCI with drug-eluting stents was non-inferior to CABG with respect to the composite of cardiac death, myocardial infarction, repeat revascularization and stroke at 1 year, even in patients with intermediate-risk according to the Syntax II score calculation.

## Figures and Tables

**Table 1:** Baseline characteristics of the patients according to the type of invasive treatment.

Variable	All Patients (n = 296)	PCI Group (n = 201) 68%	CABG Group (n = 95) 32%	*p* Value
Age, years, mean ± SD	66 ± 9.6	66.5 ± 9.9	65.4 ± 9.1	0.35
Male sex, n (%)	223 (75.6)	145 (72.1)	78 (82.1)	0.044
Hypertension, n (%)	256 (87.4)	179 (89)	77 (81)	0.108
Diabetes, n (%)	102 (34.8)	75 (37.3)	27 (28.4)	0.157
Dyslipidemia, n (%)	236 (80.5)	173 (86.1)	63 (66.3)	0.0002
Smoking, n (%)	37 (12.7)	29 (14.4)	8 (8.4)	0.162
Obesity, n (%)	72 (24.7)	58 (28.9)	14 (14.7)	0.008
Chronic kidney disease, n (%)	45 (15.4)	38 (18.9)	7 (7.4)	0.011
Prior ischemic stroke, n (%)	25 (8.6)	17 (8.45)	8 (8.42)	0.96
Prior MI—treated conservative, n (%)	50 (17.1)	32 (16)	18 (18.9)	0.48
Prior PCI				
Bare metal stent, n (%)	11 (3.8)	8 (4)	3 (3.16)	0.746
Drug-eluting stent (%)	27 (9.2)	20 (10)	7 (7.37)	0.496
Clinical presentation				
Stable angina, n (%)	179 (60.5)	95 (47.5)	83 (87.4)	
Unstable angina, n (%)	89 (30.1)	85 (42.5)	4 (4.2)	<0.0001
NSTEMI, n (%)	18 (6.1)	13 (6.5)	5 (5.3)	
STEMI, n (%)	10 (3.4)	7 (3.5)	3 (3.1)	
LV dilatation, n (%)	47 (16.3)	32 (16)	15 (15.8)	0.856
Hipokinesia, n (%)	216	154 (76.6)	62 (65.3)	0.186
LVEF on admission, %, mean ± SD	49.3 ± 8.3	49.24 ± 7.87	49.5 ± 9.18	0.808
SYNTAX II score				
0, n (%)	107 (36.1)	71 (35.3)	36 (37.9)	0.392
1, n (%)	189 (63.9)	130 (64.7)	59 (62.1)	
Other coronary artery stenosis, n (%)	172 (50.7)	105 (52.2)	75 (79)	<0.0001
No. of supplementary coronary stenoses				
1, n (%)	76 (26.4)	48 (24)	28 (29.5)	
2, n (%)	40 (13.9)	20 (10)	20 (21)	0.09
3, n (%)	52 (18.1)	41 (20.4)	11 (11.6)	
4–5, n (%)	7 (2.4)	7 (3.5)	7 (7.4)	
Complete revascularization, n (%)	189 (63.9)	138 (68.7)	51(53.7)	0.012
LVEF on follow-up, %, mean ± SD	49.7 ± 7.1	47.4 ± 8.4	52 ± 5.8	0.0001
Follow-up event				
Cardiac death, n (%)	18 (6.1)	9 (4.5)	9 (9.5)	
MI, n (%)	18 (6.1)	13 (6.5)	5 (5.3)	0.002
Stroke, n (%)	7 (2.4)	3 (1.5)	4 (4.2)	
Repeat revascularization, n (%)	29 (9.8)	12 (6)	17 (17.9)	

CABG, coronary artery bypass grafting; LV, left ventricular; LVEF, left ventricular ejection fraction; MI, myocardial infarction; NSTEMI, non-ST-elevation acute myocardial infarction; PCI, percutaneous coronary intervention; STEMI, ST-elevation acute myocardial infarction.

## Data Availability

Not applicable.
